# A rare case of malignant solitary fibrous tumor in prostate with review of the literature

**DOI:** 10.1186/s13000-017-0640-5

**Published:** 2017-07-07

**Authors:** Andrea Ronchi, Elvira La Mantia, Vincenzo Gigantino, Sisto Perdonà, Marco De Sio, Gaetano Facchini, Renato Franco, Annarosaria De Chiara

**Affiliations:** 1 Pathology Unit, Università della Campania “L. Vanvitelli”, Via Luciano Armanni, 80138 Naples, Italy; 2 Pathology Unit, Istituto Nazionale Tumori I. R. C. C. S. “Fondazione Pascale”, Naples, Italy; 3 Division of Urology, Department of Uro-Gynaecological Oncology, Istituto Nazionale Tumori I. R. C. C. S. “Fondazione Pascale”, Naples, Italy; 4 Division of Urology, Università della Campania “L. Vanvitelli”, Naples, Italy; 5 Division of Medical Oncology, Department of Uro-Gynaecological Oncology, Istituto Nazionale Tumori I. R. C. C. S. “Fondazione Pascale”, Naples, Italy

**Keywords:** Mesenchymal neoplasm, Prostate neoplasm, Spindle cell tumor, Haemangiopericytoma, Soft tissue tumor

## Abstract

**Background:**

Solitary fibrous tumor is an uncommon soft tissue neoplasm with intermediate biological behavior, which rarely metastasizes. Malignant solitary fibrous tumor, although not clearly defined, is rarely described in the prostate. The present case is characterized by some peculiarities if compared with previously reported cases of prostatic malignant solitary fibrous tumor. Firstly, it does not show a homogeneous morphology: part of the neoplasm (about 50%) showed the features of a conventional solitary fibrous tumor, while the remaining part showed the features of a malignant solitary fibrous tumor. In addition, the case is the first malignant solitary fibrous tumor reaching a huge diameter of 20 cm and replacing all prostatic parenchyma. Interestingly, normal prostatic parenchyma was observed on left-lobe trans-rectal needle-core biopsies, but was totally absent in surgical specimen. Since radical prostatectomy was carried out about 4 months after the biopsies, such discordant data may suggest exceedingly rapid growth of the neoplasm.

**Case presentation:**

We report a case of a 62-year-old male, presented at medical observation for urinary retention, constipation and an enlarged prostate gland. A trans-rectal prostatic biopsy showed a low-grade spindle cell neoplasm. Histopathological examination of the prostatectomy specimen showed patternless architecture with hypocellular and hypercellular areas and hemangiopericytoma-like vessels. In some fields the neoplasm was characterized by a high mitotic index and evident cellular atypia. Immunohistochemically, neoplastic cells were positive for CD34, bcl2, CD99, STAT6 and partially for PgR. The neoplasm was diagnosed as a malignant solitary fibrous tumor.

**Conclusions:**

The differential diagnosis of spindle cells tumors arising in the prostrate is broad and includes lesions of epithelial and mesenchymal origin, primary prostatic lesions such as stromal tumors of uncertain malignant potential and stromal sarcoma, as well as anatomically ubiquitous soft tissue neoplasms. Solitary fibrous tumors should be considered in cases of prostatic tumors with a spindled morphology, but malignancy in such tumors is extremely rare in the prostate. A review of literature showed only four additional cases. Because of the unpredictable biological behavior and the possibility of recurrence, a long-term clinical and instrumental follow-up is recommended.

## Background

Solitary fibrous tumor (SFT) is a rare mesenchymal neoplasm of postulated myofibroblastic origin [1]. The definitive etiology of SFT remains unknown, but some pathogenic mechanisms have been described. In fact, the tumor is often associated with *NAB2-STAT6* gene fusion, arising from recurrent intra-chromosomal rearrangements on chromosome 12q13 [2]. This genetic alteration deregulates the expression of *NAB2*, an important regulator of the transforming growth factor β (TGF β), and *STAT6* (signal transducer and activator of transcription), a transcriptional factor modulating signaling through interleukin-4 and interleukin-13. The genetic fusion can occur at different breakpoints and distinct fusion types may be associated with distinct clinic-pathologic subgroups of SFT [3]. The phenotypical effect of this rearrangement is nuclear expression of the C-terminal portion of STAT6 [4]. SFT is labeled as an intermediate, rarely metastasizing, neoplasm with variable clinical behavior [1]. A more aggressive variant is malignant SFT, with higher rates of local recurrences and distant metastasis [5]. Despite wide surgical excision, estimated recurrence rate for malignant SFT is approximately 30% [5]. A recently described entity is dedifferentiated SFT, which shows abrupt transition from conventional SFT to a high-grade sarcoma [6]. Wide surgical excision is the most important prognostic factor and prognosis is worse in patients with metastatic disease, with median survival ranging from 22 to 46 months [5]. For unresectable disease, chemotherapy and radiation therapy showed variable efficacy [7].

## Case presentation

**Fig. 1:**
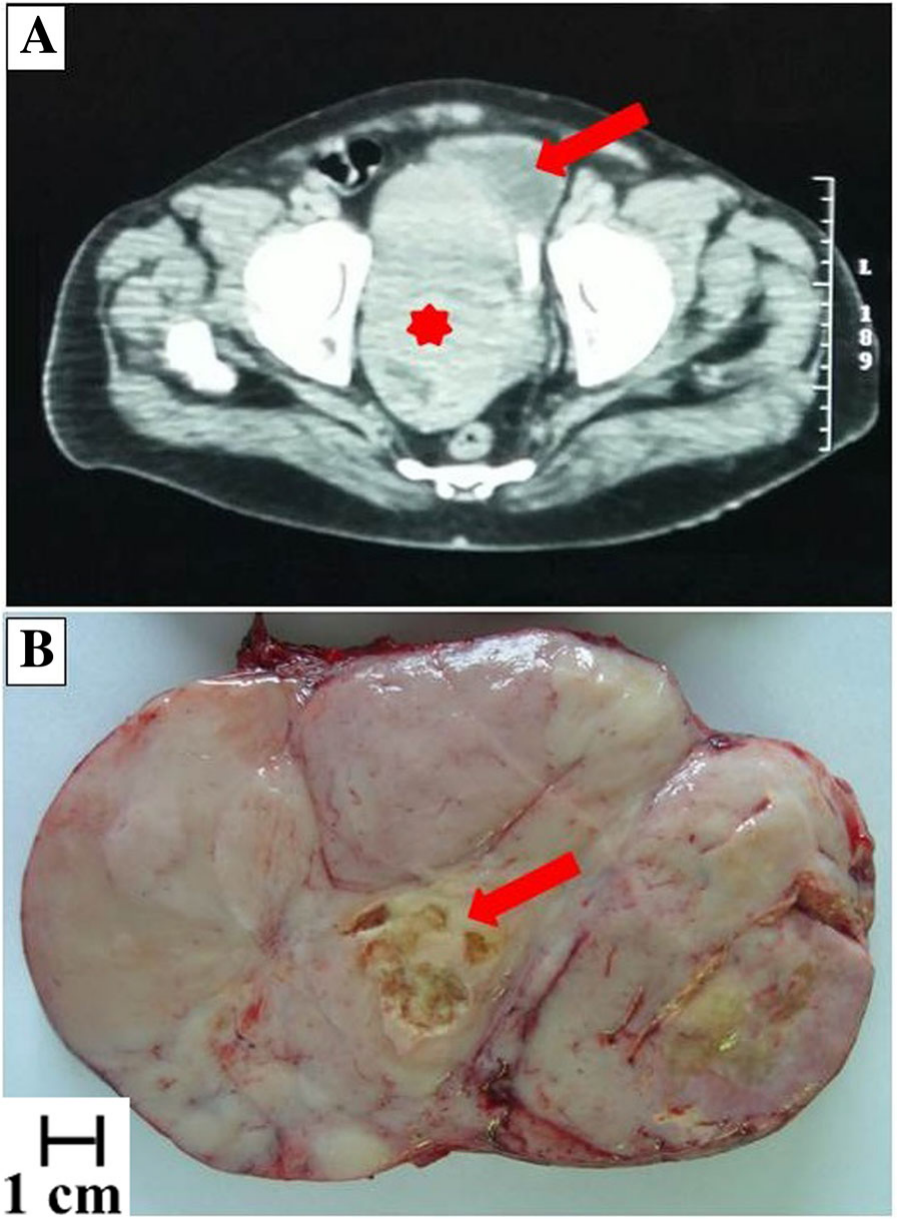
Computed Tomography and macroscopic features of the neoplasm. **a** Computed tomography showed a large, well defined mass compressing urinary bladder (*asterisk marks* the prostatic mass; *arrow marks* the urinary bladder); **b** Gross appearance of the surgical specimen showing lobulated, well defined and whitish mass with central area of necrosis (*arrow*)

**Fig. 2:**
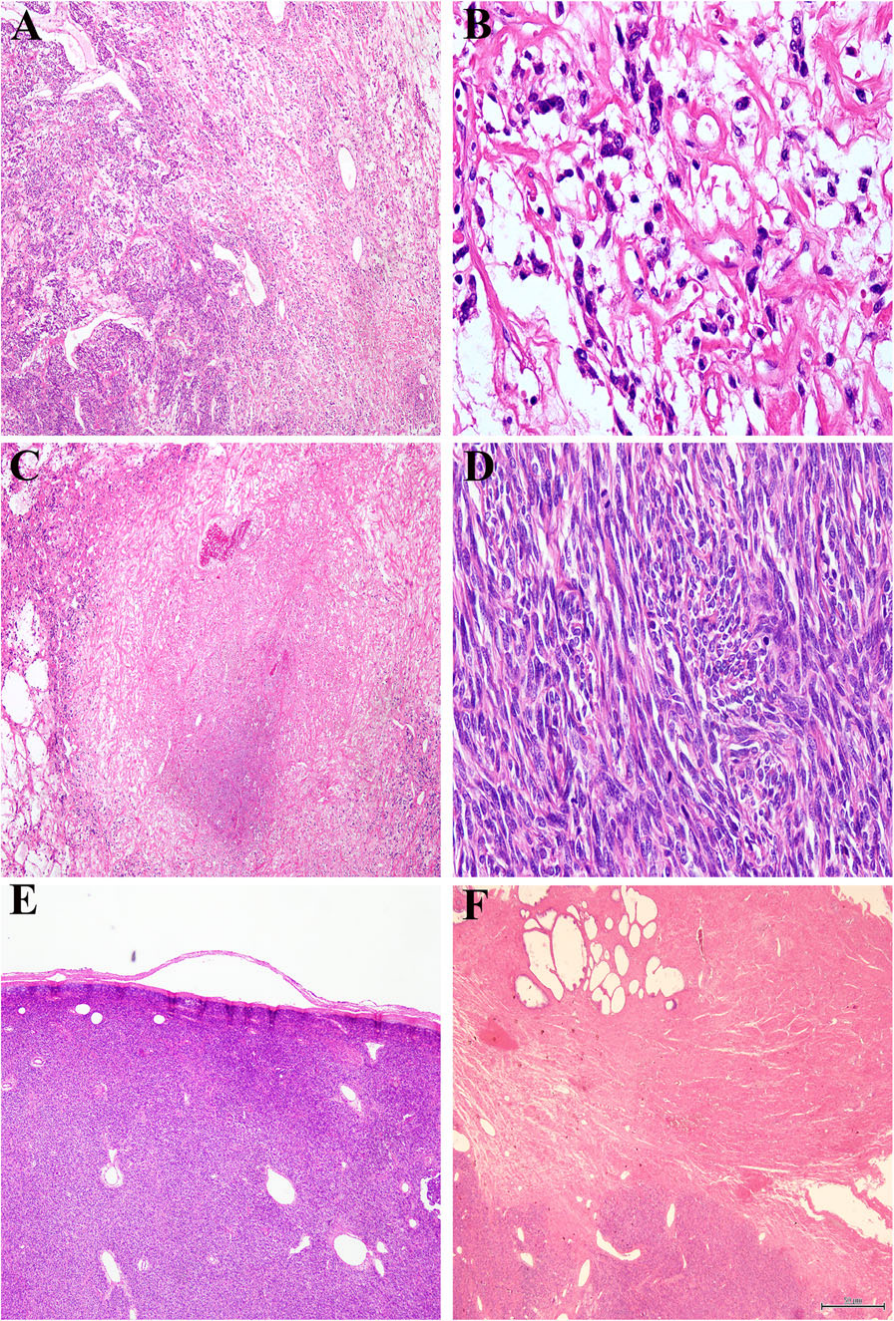
Histological features of the neoplasm. (**a** alternating hypocellular and hypercellular areas; **b** hypocellular area with a patternless population of ovoid cells with bland nuclear features; **c** area of necrosis; **d** hypercellular area with short compact fascicles of spindle cells with moderately atypical nuclei and some evident mitotic figures; **e** well-demarcated border; **f** infiltrating behavior in proximity to the right seminal vesicle)

**Fig. 3:**
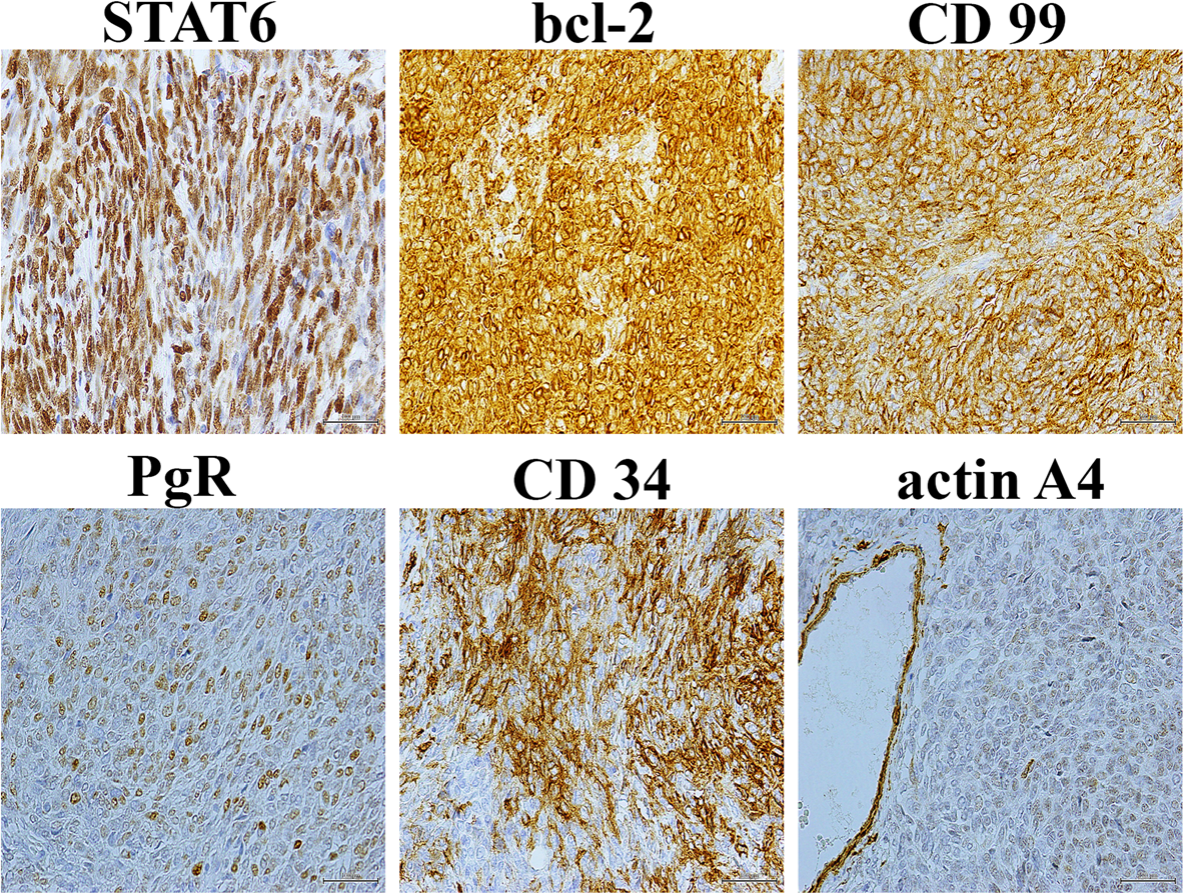
Immunohistochemical features of the neoplasm. Neoplastic cells are diffusely positive for STAT6, bcl-2, CD99 and CD34; partially positive for PgR and negative for actin A4

A 62-years-old man was admitted to the National Cancer Institute of Naples with urinary retention and lower urinary tract symptoms. Serum prostate-specific antigen (PSA) level was 5.80 ng/ml. The prostate was enlarged, smooth and firm on digital rectal examination, and a trans-rectal needle core biopsy was planned. Histologically, the tissue from the left lobe showed normal prostatic parenchyma, while cores rom the right lobe were completely occupied by a patternless neoplastic proliferation of ovoid and spindle cells with ill-defined cellular borders and relatively homogeneous nuclei, embedded in a variable quantity of fibrous stroma. Irregularly shaped, “hemangiopericytoma-like” vessels were evident, while neither necrotic areas nor mitosis were present. Immunohistochemical examination revealed that the neoplastic cells were positive for CD34 and bcl2, partially positive for progesterone receptor (PgR) and negative for cytokeratin (CK), PSA, smooth muscle actin (SMA), calponin and CD117. A diagnosis of low grade sarcoma was made, deferring the final diagnosis to the surgical specimen. Computed Tomography (CT) of the pelvis revealed a huge lobulated mass of about 20 × 10 cm involving the prostate gland and pushing the bladder base. The patient underwent radical prostatectomy about 4 months after the biopsy. Gross evaluation of the surgical specimen revealed a well-defined, whitish and lobulated mass with a central area of necrosis (Fig. 1). Histologically, the neoplasm presented variable appearance with alternating hypocellular and hypercellular areas. Hypocellular areas showed a patternless population of ovoid cells and a large number of irregular blood vessels. The central area of the neoplasia was hypercellular with spindle cells forming short compact fascicles arranged in a whirling pattern, moderately atypical nuclei and some evident nucleoli. In this area, about 8 mitosis per 10 high power fields and the presence of necrosis were evident. Interestingly, no residual prostatic parenchyma was found. The tumor was generally well demarcated, but showed infiltrating behavior close to the right seminal vesicle; the margins of surgical resection, however, were free of neoplasia. Histological features of the neoplasm are summarized in Fig. 2. Immunohistochemically, the tumor was positive for vimentin, CD34, CD99 and bcl2 while it was immunonegative for CK, SMA, S100, Epithelial Membrane Protein (EMA), desmin, calponin, CD10, CD117, estrogen receptor (ER), B-catenin. About 40% of neoplastic cells stained for progesterone receptor (PgR). A final diagnosis of malignant solitary fibrous tumor was made. Some years later, in the course of the re-examination of a series of SFTs from our files, the diffuse STAT6 nuclear positivity in the present case further confirmed the diagnosis. Immunohistochemical features of the neoplasm are summarized in Fig. 3. No adjuvant therapy was administered after surgery and no recurrences were recorded in a follow up 8 years later.

## Discussion

The onset of a malignant SFT is an exceedingly rare event, especially in the prostate, and diagnosis can be challenging, as most of the prostatic mesenchymal neoplasms are characterized by spindle cell morphology with frequent overlapping in histological features. Differential diagnosis includes a broad variety of lesions, both histotypes restricted to the prostate and anatomically ubiquitous soft tissue neoplasms [8]. It is particularly difficult to differentiate prostatic malignant SFT from prostatic stromal sarcoma (PSS) and gastrointestinal stromal tumors (GIST). PSS is a spindle cell neoplasm, which may have storiform, epithelioid or fibrosarcomatous pattern. The neoplasm is characterized by stromal overgrowth with hypercellularity and a variable degree of necrosis, cytologic atypia and mitotic activity [9]. The stromal component can be solid or mixed with benign glandular elements. Primary prostatic GIST has been rarely reported in literature, and more frequently the neoplasm arises from the rectum or perirectal space with subsequent involvement of the prostate [10, 11]. Histologically, GIST shows spindle cells with bland nuclei and perinuclear vacuoles, organized in a fascicular growth pattern. A correct pre-operative diagnosis of GIST involving the prostate is particularly important because of the possibility of medical treatment with tyrosine kinase inhibitors, compared with the surgical treatment of most other prostatic mesenchymal lesions [12]. The appropriate use of immunohistochemistry is always necessary for an accurate diagnosis. In this setting, the most helpful markers are SMA, desmin, CK, CD117, STAT6, CD34, PgR, bcl 2 and CD99. Immunohistochemical stainings in prostatic mesenchymal lesions is summarized in Table 1. PgR is frequently expressed in PSS, supporting its derivation from the hormonally responsive prostate mesenchyme [13, 14]. However PgR expression has been variably described in other prostatic mesenchymal neoplasms including smooth muscle tumours and SFT [15, 16]. Diagnosis of SFT has classically been based on the immunohistochemical expression of markers such as CD34, bcl2 and CD99. Nevertheless, recent studies have demonstrated the poor specificity of these markers [17]. CD34 is expressed significantly in a large number of soft tissue neoplasms and many entities that are included in the differential diagnosis of SFT, such as PSS and GIST, share CD34 expression [11]. Furthermore CD34 expression is absent in approximately 5–10% of conventional SFT and in the large majority of malignant and dedifferentiated forms [18, 19]. Therefore the absence of CD34 expression does not exclude the diagnosis of SFT. The expression of Bcl2 and CD99 are variably used to support the diagnosis of SFT but are less sensitive than CD34 and equally non-specific [17]. Recently, the discovery of the *NAB2-STAT6* fusion gene in SFT led to development of a STAT6 antibody that is a reliable immunohistochemical marker with a high level of sensitivity and specificity [4–20]. Therefore, nuclear expression of STAT6 is currently the most useful marker for distinguishing SFT from its histologic mimics. STAT6 nuclear expression has been demonstrated both in conventional and in malignant SFT, while dedifferentiated forms frequently lose STAT6 expression [21]. Remarkably, the presence of the chimeric fusion gene *NAB2-STAT6* is not demonstrable by in-situ hybridization (ISH) methods, due to the natural proximity of the two genes [22]. Genetic studies have recently established distinct *NAB2-STAT6* fusion gene variants associated with different clinical behavior. In particular, *NAB2* exon 6 – *STAT6* exon 16/17/18 variants seem to be associated with malignant morphology and aggressive clinical course, while *NAB2* exon 4 –*STAT6* exon 2/3 variants are associated with pleural origin and less aggressive clinico-pathological features [23]. Recently, immunohistochemical expression of ALDH1 (Aldehyde Dehydrogenase 1), an intracytoplasmatic enzyme highly expressed in stem cells, has been demonstrated in SFT [24, 25]. In one study, cytoplasmic ALDH1 expression was shown to be positive in 76% of SFTs [25]. Thus, the use of both STAT6 and ALDH1 immunostaining increases significantly the specificity of SFT diagnosis especially in the differential diagnosis with stromal tumor of uncertain malignant potential (STUMP) and SS [24]. SFT is classified in the 2013 edition of the World Health Organization (WHO) classification of tumors of soft tissue as a fibroblastic/myofibroblastic neoplasm with intermediate-rarely metastasizing biological behavior [1]. However the clinical behavior of individual tumors is not easily predictable. A clinicopathological study of 110 SFT cases by Demicco et al. elaborated a risk stratification model including age, tumor dimension and mitotic figures. Thus, SFT patients with higher risk of progression were characterized by tumor size larger than 15 cm, age higher than 55 years and mitotic figures more than 4/10 high-power fields [26]. According to this model, our patient had the highest risk score; nevertheless, no recurrence or metastases were found in a follow up 8 years later.

**Table 1:** Immunohistochemical features of spindle cell lesions of the prostate

	pankeratin	SMA	Desmin	Myogenin	CD34	PSA	PgR	CD117	STAT6
STUMP	neg	neg/pos	neg/pos	neg	pos	neg	pos	neg	neg
PSS	neg	neg/pos	neg/pos	neg	pos	neg	pos	neg	neg
SMT	neg/pos	pos	pos	neg	neg	neg	pos/neg	neg	neg
IMT	neg/pos	pos	pos	neg	neg	neg	neg	pos/neg	neg
SFT	neg	neg/pos	neg	neg	pos	neg	pos/neg	neg	pos
GIST	neg	pos/neg	pos/neg	neg	pos	neg	neg	pos	neg
SC	pos/neg	neg/pos	neg/pos	neg	neg	pos/neg	neg	neg	neg

*STUMP* stromal tumor or uncertain malignant potential, *PSS* prostatic stromal sarcoma, *SMT* smooth muscle tumors, *IMT* inflammatory myofibroblastic tumor, *SFT* solitary fibrous tumor, *GIST* gastrointestinal stromal tumor, *SC* sarcomatoid carcinoma, *RMS* rhabdomyosarcoma, *SMA* smooth muscle actin, *CD34* cluster of differentiation 34, *PSA* prostate-specific antigen, *PgR* progesterone receptor, *CD117* cluster of differentiation 117, *STAT6* signal transduction and activation of transcription 6

While several cases of conventional SFT have been reported in the prostate, the onset of malignant SFT in the prostate is an extremely rare event and the review of the literature reveals only four other cases. In 2005 Vodovnik et al. described a case of malignant SFT in a 87 years-old man [27]. The patient presented massive haematuria and died on the first post-operative day following a cardiac arrest. Surgical specimen consisted of five nodular pieces of prostatic parenchyma, measuring 2–9 cm in greatest diameter. Three other cases were further described by Herawi and Epstein in 2007 [28]. These cases were diagnosed on either needle biopsies or transurethral resections of the prostate. Follow-up was available for two patients showing that both patients were alive and without any evidence of disease at 5 and 10 years after the initial diagnosis. Unfortunately other clinical details were not described by the authors. The case we present is also characterized by a particular clinical behavior. This is the first described case of malignant SFT, which reaches a huge diameter of 20 cm and replaces the whole prostatic parenchyma. Interestingly, normal prostatic parenchyma was present on left-lobe trans-rectal needle-core biopsies, but it was totally absent in surgical specimen. Since radical prostatectomy was performed about 4 months after the biopsies, this peculiarity may suggest an exceeding rapid growth of the neoplasm.

## Conclusions

SFT is a rare neoplasm of mesenchymal origin that should be considered in cases of prostatic tumor with a spindled morphology. Positive nuclear staining for STAT6 is currently the most accurate diagnostic test. Considering the low predictable biological behavior and the possibility of recurrence and metastases, a long-term clinical and instrumental follow-up is recommended.

## Abbreviations

ALDH1: Aldehyde dehydrogenase 1
bcl2: b-cell lymphoma 2
CD: Cluster of differentiation
CK: Cytokeratin
CT: Computed tomography
EMA: Epithelial membrane protein
ER: Estrogen receptor
GIST: Gastrointestinal stromal tumor
IMT: Inflammatory myofibroblastic tumor
ISH: in-situ hybridization
ml: milliliters
ng: nanograms
PgR: Progesterone receptor
PSA: Prostate-specific antigen
PSS: Prostatic stromal sarcoma
RMS: Rhabdomyosarcoma
SC: Sarcomatoid carcinoma
SFT: Solitary fibrous tumor
SMA: Smooth muscle actin
SMT: Smooth muscle tumors
STAT6: Signal transducer and activator of transcription 6
STUMP: Stromal tumor of uncertain malignant potential
TGF β: Transforming growth factor β
WHO: World Health Organization
